# Clinical features of hepatolithiasis: analyses of multicenter-based surveys in Japan

**DOI:** 10.1186/s12944-015-0130-2

**Published:** 2015-10-17

**Authors:** Susumu Tazuma, Yasuni Nakanuma

**Affiliations:** Department of General Internal Medicine, Hiroshima University Hospital and Graduate School of Biomedical & Health Sciences, Hiroshima, Japan; Department of Human Pathology, Kanazawa University School of Medical Sciences, Kanazawa, Japan

**Keywords:** Hepatolithiasis, Cholangiocarcinoma, Balloon endoscopy

## Abstract

**Background:**

Hepatolithiasis is a calculus disease of the liver with no known cause that is relatively uncommon, and is characterized by a refractory nature and high frequency of recurrence. Hepatolithiasis is one of the diseases listed by the Ministry of Health, Labour and Welfare of Japan under Research on Intractable Diseases, and it requires further research on the pathogenesis as well as the therapeutic strategy. It is also included in the clinical guidelines for cholelithiasis of the Japanese Society of Gastroenterology, which suggest guiding principles for the treatment of hepatolithiasis.

**Methods:**

we performed questionnaire surveys of hepatolithiasis twice in 2010 and in 2012. Verification of the evidence-based clinical practice guidelines a questionnaire survey of 22 facilities in 2010 and 25 facilities in 2012 across Japan that provided cooperation, which enabled us to assess 210 new cases over a two-year period.

**Conclusions:**

Comparison with two surveys that have been carried out previously revealed the main factor associated with hepatolithiasis was a history of biliary tract surgery, which was noted in the majority of cases. In addition, there was an increase of patients in whom balloon endoscopy was performed using transduodenal approach. This method is not included in the treatment options of the current clinical guidelines for cholelithiasis, so there may be a need to take it into consideration when the guidelines are revised.

## Introduction

Hepatolithiasis occurs less frequently compared to other stone diseases of the biliary tract, such as cholelithiasis and choledocholithiasis. The nationwide, cross-sectioned surveys in Japan demonstrates the incident of hepatolithiasis accounts for 1.3 % of all cholelithiasis cases in 1997 and about 0.6 % in 2006. Its pathology, including the causes of calculus formation, is complex, and the disease shows a refractory and recurrent nature. Accordingly, this disease has been studied as part of the research projects on intractable diseases of the Ministry of Health, Labour and Welfare of Japan since 1978. In 2009, the clinical guidelines for cholelithiasis edited by the Japanese Society of Gastroenterology were published, which also cover the diagnosis and treatment of hepatolithiasis [1].

Currently, hepatectomy, endoscopic mechanical lithotripsy, and shock wave lithotripsy are performed alone or in combination for the treatment of hepatolithiasis, but the indications for selecting these treatment methods have not been established at present. In addition, it is a characteristic of this disease that patients frequently develop conditions such as bile duct dilatation after undergoing biliary tract surgery, and many of these patients encounter postoperative problems after reconstruction. However, no clear therapeutic strategies have been suggested for patients with a surgical history.

Furthermore, while the presence of bile duct stenosis has been suggested as a cause of lithogenesis and recurrence, bile duct stenosis and regions of hepatic atrophy are also viewed as sites from which bile duct cancer arises. Thus, apart from being a risk factor for lithogenesis and recurrence, it is believed that monitoring sites of stenosis is the key element during follow-up after calculus removal.

In the present study, we asked various facilities across Japan to participate in a questionnaire survey and evaluated the responses in order to investigate the recent incidence, methods of diagnosis, and therapeutic approach to hepatolithiasis, with the aim of examining the validity of the current clinical guidelines.

## Methods

The present survey was conducted by sending questionnaires regarding new hepatolithiasis patients over a two-year period to facilities associated with the Japan Biliary Association or the Japanese Research Committee on Intractable Hepatic and Biliary Diseases supported by the Health and Labor Sciences Research Grant (Research on Intractable Diseases) of the Ministry of Health, Labour and Welfare of Japan.

The questionnaire required responses regarding new patients encountered between 2010 and 2011. Similar surveys have already been performed between 2006 and 2007 and between 2008 and 2009. In 2009, the clinical guidelines for cholelithiasis edited by the Japanese Society of Gastroenterology were published, which also cover the diagnosis and treatment of hepatolithiasis [1], so two questionnaire surveys between 2008 and 2009, and between 2010 and 2011, were in principle compared in this study. Participants were asked to provide responses to the following six items: 1) the number of cases, 2) the presence or absence of symptoms, 3) history of biliary tract surgery, 4) methods of diagnosis, 5) therapeutic approach, and 6) prognosis and complications. The number of facilities that provided responses was 22 in the survey between 2008 and 2009, and 25 in the survey between 2010 and 2011. To assess the changes of clinical features of management of hepatolithiasis, the number of patients per facility, the presence and absence of symptoms, history of biliary tract surgery, diagnostic modalities such as ultrasound and/or X-ray (US/XP), computed tomography and/or magnetic resonance cholangiopancreatography (CT/MRCP), drip infusion cholangiography-CT and/or endoscopic retrograde cholangiopancreatography and/or endoscopic ultrasound (DIC-CT/ERCP/EUS), treatment approaches such as surgical or nonsurgical treatment or others, and prognosis and complications were evaluated.

## Results

### Number of patients

**Fig. 1:**
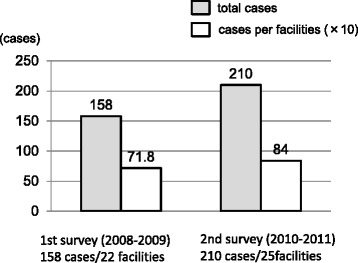
Number of cases

The total number of patients and facilities in the first and second surveys was 158 patients/ 22 facilities, and 210 patients/ 25 facilities, respectively. The number of patients per facility in the first and second surveys was 7.2 and 8.4, respectively (Fig. 1). Thus, both of the total number of patients and patients per facility were increased in the second survey when compared to the first survey.

### Symptoms

**Fig. 2:**
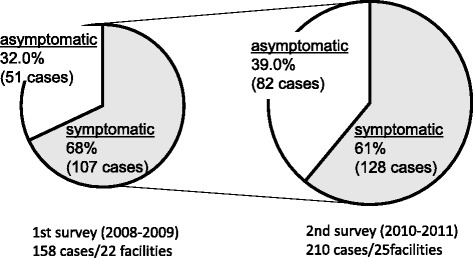
Presence of symptoms

In both the first and second surveys, patients presenting with symptoms accounted for 68 % and 61 % of all cases, whereas asymptomatic patients accounted for 32 % and 39 %, respectively (Fig. 2). In both surveys, patients with symptoms were predominant.

### History of biliary tract surgery

**Fig. 3:**
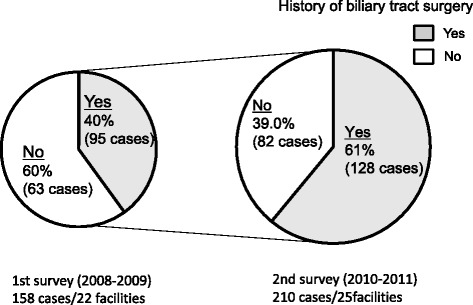
History of biliary tract surgery

Patients with a history of surgical procedures on the biliary tract accounted for 40 % in the first survey, and increased to 61 % in the second survey (Fig. 3). There was an increase of treatment for patients with hepatolithiasis who had previously undergone biliary surgery (data not shown).

### Diagnosis

**Fig. 4:**
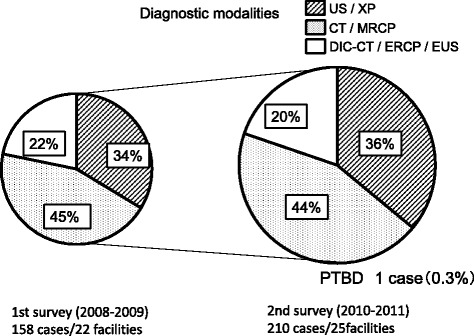
Diagnostic modalities

This question concerned the actual examination methods that led to the diagnosis of hepatolithiasis. In both of the first and second surveys, US/XP accounted for approximately 34 ~ 36 %, CT/MRCP for approximately 44 ~ 45 %, and DIC-CT/ERCP/EUS for approximately 20 ~ 22 % (Fig. 4). No drastic changes of the diagnostic methods were observed.

### Treatment

**Fig. 5:**
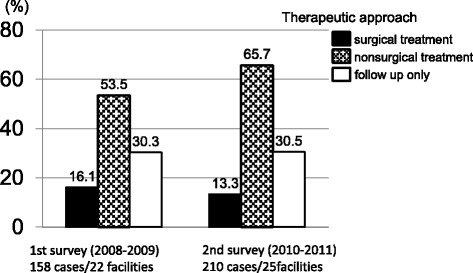
Therapeutic approach

**Fig. 6:**
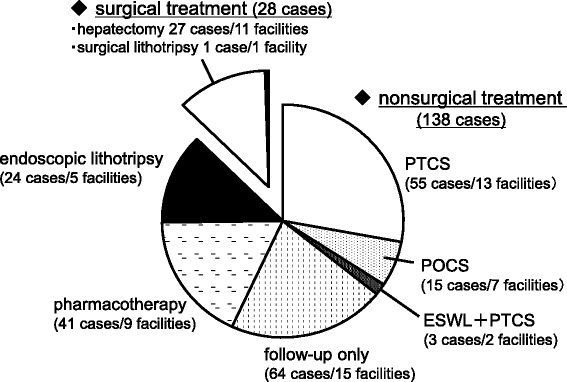
Trends of therapeutic approach (2010–2011)

We asked about selection of the therapeutic approach to hepatolithiasis. Surgical treatment, including hepatectomy, intraoperative biliary fiberscopy, laparoscopic choledochotomy, choledochojejunostomy, and liver transplantation, was performed for 16.1 % in the first survey and 13.3 % in the second survey, so there was a slight decreasing trend (Fig. 5). On the other hand, nonsurgical treatment, including percutaneous transhepatic cholangioscopy (PTCS), endoscopic transpapillary therapy (endoscopic retrograde cholangiopancreatography: ERCP, peroral cholangioscopy: POCS), extracorporeal shock wave lithotripsy (ESWL), and pharmacotherapy was 53.5 % in the first survey, and 65.7 % in the second survey, showing an increase over the two surveys (Fig. 5). As for the endoscopic route, the percentage of patients treated via the percutaneous and transpapillary approaches was about the same in each survey. With the transpapillary/ transduodenal approach, however, there was a decrease in the number of cases where lithodialysis was performed via biliary endoscopy, whereas the number of cases of direct calculus removal increased. Data for therapeutic approaches in the second survey were shown in Fig. 6. On the other hand, the percentage of patients who were followed up without undergoing calculus removal was 30.3 ~ 30.5 % (Fig. 5).

**Fig. 7:**
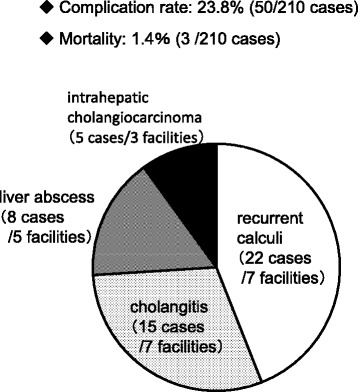
Prognosis and complications (2010–2011)

### Prognosis and complications

We asked about recurrence and complications in the early and late phases, including deaths. The complication rate was 23.8 %, and 50 out of 210 patients developed complications as shown in Fig. 7. The most frequently reported complication was recurrent calculi, followed by cholangitis, liver abscess, and intrahepatic bile duct cancer, in this order. It was reported that complications led to death in three patients including cholangitis and postoperative death (1.4 %).

## Discussion

The objectives of this survey were to examine the trend in the number of patients with hepatolithiasis, assess changes in the therapeutic approach, and confirm the validity of the clinical guidelines of the Japanese Society of Gastroenterology regarding hepatolithiasis.

Hepatolithiasis has been recognized as an intractable disease since the Research Committee on Intrahepatic Bile Duct Disorders was organized in 1978. This committee is currently known as the Research Committee on Intractable Hepatic and Biliary Diseases, Subcommittee on Hepatolithiasis, which is part of the Japanese Ministry of Health, Labor and Welfare Research on Intractable Diseases. It continues to perform investigations and studies in order to determine the causes and clinical features of hepatolithiasis, as well as to establish new treatment methods. In the first nationwide survey conducted by this study group, which collected cases between 1975 and 1984, 4,381 patients (438 per year) were reported during the 10-year period. The number of cases in the last three surveys remains 210 over 2 years (105 per year), which suggests a decreasing trend, although there were differences in the size of the hospitals surveyed.

No significant changes were observed in the recent methods used for the diagnosis of hepatolithiasis. However, we observed changes in the selection of treatment, as new therapies were reported. When selecting the treatment strategy for hepatolithiasis, the choice needs to be based on 1) the composition of the calculi (i.e., cholesterol stones or calcium bilirubinate stones); 2) the location of the calculi in order to choose an approach; 3) the planned treatments including alleviation of bile duct stenosis; 4) evaluation of liver atrophy and the extent of liver resection; and 5) evaluation of intrahepatic bile duct cancer. A therapeutic strategy is developed by also taking into consideration various factors such as a history of biliary tract surgery, in addition to the points mentioned above. According to the hepatolithiasis treatment flow chart in the 2009 clinical guidelines for cholelithiasis produced by the Japanese Society of Gastroenterology, treatment selection should be based on the presence or absence of a history of biliary tract surgery.

When we evaluated the present survey results, the percentage of postoperative cases showed an increase in the third survey. In terms of treatment, there was a slight decrease of surgical procedures and follow-up, while an increase was observed in endoscopic procedures. This trend might be due to a change in the management of postoperative patients, for whom active procedures were avoided in the past. It is likely that balloon endoscopy has been applied clinically as a non-surgical treatment in recent years, which made the transduodenal approach possible in cases where postoperative biliary reconstruction is performed. The hepatolithiasis treatment flowchart in the cholelithiasis guidelines only recommends PTCS when treating patients with a history of surgery on the biliary tract [2–5]. Although it is also noted that minimally invasive, repeatable treatment methods centering on PTCS can be selected in combination with other procedures such as ESWL, the results of the present survey have suggested the usefulness of transduodenal procedures employing balloon endoscopy [6]. Given the clinical position of this method, we think that revision of the guidelines is warranted, such as adding this method to the flowchart.

In the present questionnaire survey, the responses to the question regarding a history of biliary tract surgery showed that 60 % of patients had a surgical history, making up a majority of the subjects. In addition, there was an increase in the number of cases where a transpapillary or transduodenal approach was used, which was likely to be the result of actively conducting calculus removal in patients with a surgical history. On the other hand, the percentage of hepatolithiasis complicated by intrahepatic bile duct cancer has been reported to be 4.0–12.5 % [7–13], and it has been pointed out that the risk of developing cholangiocarcinoma after removal of calculi is increased by the presence of bile duct stenosis [14]. In the study reports from the Health and Labor Sciences Research Grant (Research on Intractable Diseases) Research Committee on Hepatolithiasis (Atomi group) between 2005 and 2007, two factors that reduced the risk of bile duct cancer were hepatectomy and administration of UDCA. The fact that there has been an increase in the number of patients undergoing endoscopic removal of calculi suggests that there will be a need for careful follow-up, since the bile ducts from which cancer arises are preserved. Furthermore, continued administration of UDCA will be an essential requirement. When the cholelithiasis guidelines are revised, due consideration of these issues will be required.

Hepatolithiasis has been considered to be endemic in East Asia, but is occasionally observed in Western countries, especially in those who have lived in the Orient [15]. Since intrahepatic brown stones are pigment stones consisting of bilirubin and cholesterol, biliary infection with bile duct stricture and cholesterol metabolism disorders are considered to be causative factors. However, the present data of increased cases with a history of biliary surgery suggest considerable changes in the pathogenesis. Thus, it is recommended to pay attention to screening hepatolithiasis using diagnostic modalities demonstrated in guidelines through a follow-up of subjects after biliary surgery, world-widely.

In conclusion, our three questionnaire surveys have revealed that there has been an increase of hepatolithiasis patients with a history of surgical procedures on the biliary tract. In addition, an increasing number of these patients are undergoing calculus removal via the transduodenal approach with a balloon endoscope. Because the risk of bile duct cancer is increased due to preservation of the hepatic parenchyma and biliary tree, the need for follow-up or continuation of UDCA administration is expected. When the clinical guidelines for cholelithiasis are revised, the creation of a flow chart that takes these issues into account is desired.
